# Can we use as a marker the maternal serum levels of D-dimer and fibrinogen to predict intra uterin growth restriction?

**DOI:** 10.4274/tjod.39260

**Published:** 2014-12-15

**Authors:** Kerem Tetik, Kerem Doğa Seçkin, Fatih Mehmet Karslı, Seval Sarıaslan, Bülent Çakmak, Nuri Danışman

**Affiliations:** 1 Zekai Tahir Burak Womens Health Teaching and Research Hospital, Clinic of Obstetrics and Gyncology, Ankara, Turkey; 2 Tokat Gaziosmanpaşa Universty Faculty of Medicine, Department of Obstetrics and Gyncology, Tokat, Turkey

**Keywords:** IUGR, D-dimer, Fibrinogen

## Abstract

**Objective:**

The purpose of the present study was to assess the effect of D-dimer and fibrinogen levels in maternal serum as a marker for detection of intrauterine growth restriction (IUGR).

**Materials and Methods:**

Two hundred-thirty four pregnant women who get pregnancy follow-up and delivery in the tertiary hospital between January 2011 and December 2011 were admitted to the study. Pregnant women were divided into two groups; group-1: 117 pregnants complicated with IUGR and group-2: 117 healthy pregnants without IUGR as control. Serum D-dimer and fibrinogen levels of all pregnant women were measured in the third trimester. The levels of D-dimer and fibrinogen were compared between two groups.

**Results:**

There is no significant difference between the groups for age, body mass index before pregnancy, smoking and gestational weeks (p>0.05). Gravidity, parity, number of children and maternal serum fibrinogen level were detected significantly different between the groups (p<0.001). D-dimer level was not significantly different between the groups (p=0.183), but fibrinogen level in group-1 was found higher than group-2.

**Conclusion:**

Serum fibrinogen level was found higher in pregnant women complicated with IUGR but D-dimer level was not different between the groups. Although serum D-dimer should not be used as a marker for detection of IUGR, serum fibrinogen may be used.

## INTRODUCTION

Normal fetal growth depends on maternal, fetal, placental, and external factors, as well as the genetically predetermined growth potential^(1)^. The impairment of one or more of these factors affects fetal growth. The impairment in any one of these factors or a combination thereof may cause poor pregnancy outcomes and intrauterine growth restriction (IUGR)^(2)^. Intrauterine growth restriction is considered one of the important causes of perinatal mortality and morbidity in modern obstetrics practice. IUGR is the second most important cause of perinatal deaths after preterm birth^(3)^. The objective of modern perinatology should be to provide proper follow-up, timely intervention, and early detection the fetuses with IUGR to avoid further fetal damage. The American College of Obstetricians and Gynecologists (ACOG) defines IUGR as the estimated birth weight being lower than the 10^th^ percentile for gestational age. As specified in the definition, the incidence is accepted as 10%^(4,5)^. In addition to anamnesis and clinical examination, fetal ultrasonographic parameters (biparietal diameter, abdominal circumference, femur length, estimated fetal weight, head circumference/abdominal circumference, and umbilical artery Doppler) are used for diagnosis^(6)^. Abnormal uterine artery Doppler findings have been reported to be a good marker to determine preeclampsia and IUGR in the later stages of pregnancy; however, it is still not used in obstetrics practice^(7)^. Placental factors are not the most common reason, but are important in the development of IUGR^(8)^. The placental histological abnormalities defined to be associated with IUGR may be listed as abnormalities in uteroplacental vasculature, chronic villitis of unknown etiology, infraction, distal villous hypoplasia, massive previllous fibrin deposition and thrombosis in uteroplacental, and intervillous and/or fetoplacental vasculature^(9,10)^. During pregnancy, some changes also occur in the coagulation hemostatic system along with increasing weeks of pregnancy^(11)^. There is an increase in most of the procoagulant factors in the cascade (factors 1, 7, 8, 9, and 10). The plasma level of fibrinogen starts to increase in the first trimester and reaches to maximum levels in the third trimester^(12)^. Concordantly, the levels of D-dimer, fibrin degradation product, also show increase with the increasing pregnancy week^(13)^. The present study aimed to compare the pregnant women with IUGR with normal pregnant women in terms of levels of fibrin and D-dimer, increasing with gestational age, and to investigate if these parameters were beneficial in predicting IUGR.

## MATERIALS AND METHODS

The present study was conducted between January 2011 and December 2011 on a total of 234 pregnant women in two groups within the gestational age 28-38, who were admitted to Zekai Tahir Burak Women’s Health Training and Research Hospital, were followed-up and gave birth in this hospital. Required ethics committee approval and patient consents were obtained. The study group included 117 pregnant women diagnosed with IUGR, who stayed in the Perinatology Clinic. Among the 117 pregnant composing the control group, all pregnant women coming to the antenatal polyclinic for routine controls were listed and designated. Then, the patients to be included in sampling were determined by using a random numbers table. Thereby, the patients were chosen in a completely randomized manner based on simple random sampling. Age, number of pregnancies, outcomes of previous pregnancies, poor health habits such as smoking and drinking, pre-pregnancy body mass indices, ultrasound findings (biparietal diameter, abdominal circumference, femur length, amniotic fluid index, umbilical artery Doppler) of the patients included in the study were recorded. The pregnant women with a known fetal anomaly, chromosomal abnormality, fetal infection of multiple pregnancies and the pregnant women with early membrane rupture, preterm labor or term labor were excluded from the study. Additionally, it was confirmed that all pregnant women included in the study did not have any conditions that might increase the levels of serum markers such as thrombophlebitis, deep vein thrombosis, or pulmonary embolism. The pregnant women included in the study did not have any systemic diseases or a history of using any medications. The gestational ages of the patients included in the study were calculated using the date of last menstrual period and confirmed by the early ultrasonographic measurements. For all pregnant women included in the study, biometrical measurements were performed with a 3.5 mHz-convex probe, using PROSOUND SSD 5500 SV (ALOKA, Japan) USG machine. For all cases, estimated fetal weight (EFW) was calculated based on the Hadlock formula using the ultrasonographic biparietal diameter (BPD), head circumference (HC), femur length (FL) and abdominal circumference (AC) measurements. Amniotic fluid volume was assessed for all cases. During the ultrasound assessment, the sum of the vertical measurements of the four-quadrant largest amniotic pockets was considered as the amniotic fluid index (AFI). When the amniotic fluid index was lower than the 5^th^ percentile for gestational age, oligohydramnios was diagnosed. The patients with an estimated fetal weight lower than the 10^th^ percentile for gestational age, which was calculated by USG in first trimester, and with abnormal umbilical artery Doppler indices were diagnosed with intrauterine growth restriction. From the patients diagnosed with IUGR, the patients who were above the 95^th^ percentile were classified as “Asymmetric IUGR” and others as “Symmetric IUGR” according to the table of HC/AC ratio for pregnancy week. Additionally, the pregnant women with IUGR were divided into two subgroups as <3^th^ percentile and 3-10^th^ percentile for estimated fetal weight again. Pulse and color Doppler USG were recorded for all pregnant women included in the study by using the same machine as used on the mother in the supine position when there was no fetal movement. Sections were taken from the umbilical artery site that might be detected at an equal distance to the placenta and fetus, and the umbilical artery systole/diastole ratios were calculated. The pregnant women that were detected to have end diastolic flow loss and a reverse flow and the pregnant women who were above the 95^th^ percentile for gestational ages were defined as “abnormal umbilical artery Doppler”.

For serum analyses, the antecubital vein was used to obtain venous blood samples from the study and control groups. The serum samples were obtained from the case group in the week when the patient was hospitalized upon suspecting IUGR and therefore performing USG assessments, and finding that the fetal weight was below the 10^th^ percentile for the probable fetal weight in the current gestational age and there was abnormal artery Doppler. The venous blood samples from the pregnant women in the control group were obtained in weeks consistent with the study group of pregnant women. The blood samples were taken in tubes as 1/10 with sodium nitrate solution (0.11 mol/L) and the remaining was venous blood, and studied within 1 hour in the biochemistry laboratory of the aforementioned hospital by centrifuging for 10 minutes at 4000 rpm. The results were designated in µg/ml for D-dimer and mg/dl for fibrinogen.

The data were analyzed using SPSS for Windows 11.5 software package. The significance of the difference in mean values between the groups was evaluated by the student’s t-test, whereas the significance of the difference in median values was analyzed by the Mann-Whitney U-test if there were two independent groups and by the Kruskal-Wallis test if there were more than two groups. The categorical variables were evaluated by Pearson’s chi square or Fisher’s exact chi square test. P values <0.05 were considered statistically significant.

## RESULTS

**Figure 1:**
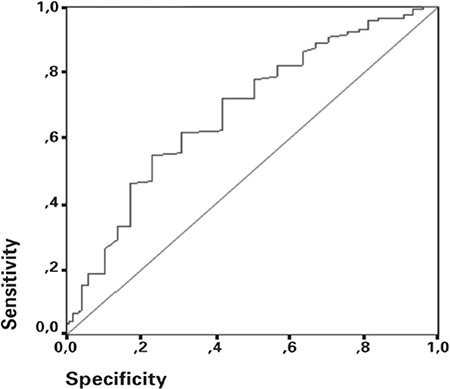
ROC curve of fibrinogen to differentiate normal group and IUGR group

In the present study, which included 234 pregnant women, 117 patients with IUGR were analyzed as group 1 and the control group with healthy pregnant women were analyzed as group 2. When the groups were compared for demographic and clinical characteristics, there was no statistical difference in maternal age, pre-pregnancy body mass index, smoking status, gravida, and gestational age. The parity and the number of living children were higher in the control group (Table 1). When the groups were compared for maternal serum levels of D-dimer and fibrinogen, there was no statistical significance despite the higher levels of D-dimer in all study groups and sub-groups; however, the fibrinogen levels were significantly higher in the study group (Table 2). When the study group was divided into subgroups by the severity of IUGR, the fibrinogen levels were significantly higher in both the groups in the <3^th^ percentile and 3-10^th^ percentile; however, there was no increase directly proportional to the severity of IUGR (Table 3). No significant difference was found in fibrinogen levels between these two groups. When the study group was again divided into subgroups as symmetric IUGR and asymmetric IUGR, the maternal serum levels of fibrinogen were significantly higher in both subgroups. When the subgroups were compared to each other, the fibrinogen levels were higher in the symmetric IUGR groups and this difference was statistically significant (Table 4). The area under the ROC curve of D-dimer and fibrinogen, the best cut-off point, and the diagnostic performance indicators under the ROC curve were used to differentiate the control group and study group; the sensitivity was 54.7% and the specificity was 76.9% for fibrinogen (Figure 1). The cut-off value was found to be 470 mg/dl for fibrinogen.

## DISCUSSION

It is important to recognize and identify IUGR and understand its etiopathogenesis since it has increased perinatal mortality, morbidity, and long-term effects^(14)^. It is important to identify and follow-up on the constitutionally small fetuses in particular among the small-for-gestational age fetuses separately from the fetuses with actual IUGR, which have a higher risk for perinatal outcome^(15)^. Normal pregnancy is a condition in which significant changes occur in the hemostatic system with an increased tendency to coagulation. These changes are considered to occur to prepare the pregnant woman for labor through the effects of hormonal mechanisms. During labor, bleeding is controlled in a quick and effective way along with uterine contraction^(16)^. The study by Hansen et al. on healthy pregnant women demonstrated that there was a statistically significant increase in D-dimer levels between the first trimester and the second trimester, and between the second trimester and the third trimester (p<0.0001). In all pregnant women studied, the D-dimer levels were above the pre-pregnancy cut-off levels in first trimester in 31% of patients, in the second trimester in 76% of patients, and in the third trimester in 98% of patients^(17)^. The fibrinogen increase was significant in the second and third trimesters (p<0.0001). In the present study, the levels of D-dimer and fibrinogen were higher in both groups compared to the pre-pregnancy values, and fibrinogen levels were statistically significant. Parallel to the present study, the study by Liu et al., which was conducted by classifying normal pregnancies based on gestational age, demonstrated that the fibrinogen levels increased significantly between weeks 13 and 20, remained relatively stable between weeks 21 and 27, started to significantly increase again between weeks 28 and 35, and remained unchanged between weeks 36 and 42^(18)^. Francalanci et al. conducted a study with a group of 37 pregnant women presenting with IUGR and a group of 37 normal pregnant women^(19)^. These pregnant women were divided into two groups as 20-30 weeks and week >30. As a result of comparing these groups, the D-dimer level was higher in the weeks 20-30 group compared to the control group in the same week (p<0.05). There was no difference in the week >30 group. Furthermore, no significant difference was found for fibrinogen in both subgroups compared to the control groups. The present study had a higher number of study and control pregnant women (n=234). Although there was a slightly higher D-dimer value between IUGR and control groups beyond week 30 in the present study, there was no statistically significant difference (1.01, 0.96 µg/ml, p=0.183, respectively). However, as distinct from the said study, the levels of fibrinogen were significantly higher in all IUGR cases compared to the control group in the present study (p<0.001). As distinct from other studies, the present study included <3^th^ percentile and 3-10^th^ percentile subgroups. The fibrinogen levels were found statistically significantly higher in both <3^th^ percentile and 3-10^th^ percentile subgroups compared to the control group. However, the fibrinogen levels did not increase as the fetal exposure increased, and no increased fibrinogen levels were observed in severe IUGR. The researchers of the current study concluded that fibrinogen that has not shown a direct proportion to the severity of IUGR cannot be an indicator for the IUGR severity. Despite the similar previous studies in the literature, no difference was found in D-dimer levels from the statistical analyses by dividing the IUGR group into symmetric (n=59) and asymmetric (n=58) subgroups. The fibrinogen level was statistically significantly higher in the symmetric group compared to the asymmetric group. Both symmetric and asymmetric groups had a higher level compared to the control group. The exposed fetus with symmetric IUGR further increased the fibrinogen levels.

In conclusion, fibrinogen is a procoagulant factor and also an acute phase reactant. D-dimer is also of fibrin origin and reflects its synthesis and degradation. The impaired maternal-fetal transports, such as IUGR, are a stress factor in this cascade and increase the maternal serum levels relative to normal pregnancy. Therefore, the maternal serum levels of fibrinogen in pregnant women with suspected IUGR may be supported with studies including more patients and may be used as a marker for predicting IUGR.

## Figures and Tables

**Table 1:**
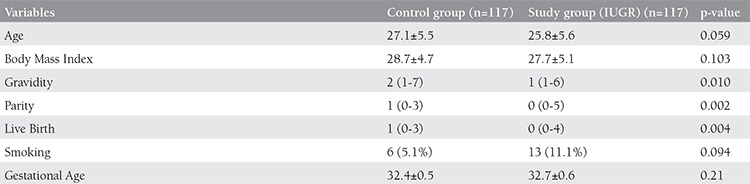
Dermographic and clinical features of control and study groups

**Table 2:**
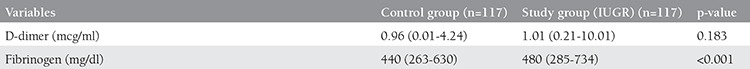
Maternal serum levels of D-dimer and fibrinogen

**Table 3:**
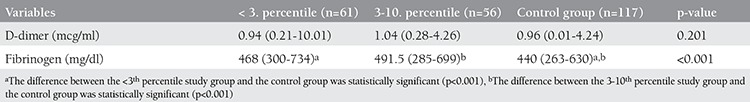
The maternal serum levels of D-dimer and fibrinogen of the <3^th^ percentile and 3-10^th^ percentile study groups compared to the control group

**Table 4:**
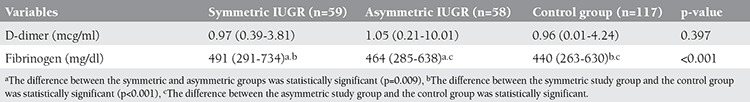
Comparing the maternal serum levels of D-dimer and fibrinogen of the symmetric and asymmetric IUGR study groups and the control group

## References

[1] Gardosi J, Mul T, Mongelli M, Fagan D (1998). Analysis of birthweight and gestational age in antepartum stillbirths. Br J Obstet Gynaecol.

[2] Baschat AA, Hecher K (2004). Fetal growth restriction due to placental disease. Semin Perinatol.

[3] Wolfe HM, Gross TL, Sokol RJ (1987). Recurrent small for gestational age birth: perinatal risks and outcomes. Am J Obstet Gynecol..

[4] Brodsky D, Christou H (2004). Current Concepts in Intrauterine Growth Restriction. J Intensive Care Med.

[5] Clausson B, Gardosi J, Francis A, Cnattingius S (2001). Perinatal outcome in SGA births defined by customized versus population-based birthweigth standarts. Br J Obstet Gynaecol.

[6] Dudley NJ (2005). A systematic review of the ultrasound estimation of fetal weight. Ultrasound Obstet Gynecol.

[7] Rotmensch S, Liberati M, Luo JS, Kliman HJ, Gollin Y, Bellati U, et al (1994). Color Doppler flow patterns and flow velocity waveforms of the intraplacental fetal circulation in growth-retarded fetuses. Am J Obstet Gynecol.

[8] Heinonen S, Taipale P, Saarikoski S (2001). Weights of placentae from small-for-gestational age infants revisited. Placenta.

[9] Salafia CM (1997). Placental pathology of fetal growth restriction. Clin Obstet Gynecol.

[10] Redline RW (2008). Placental pathology: a systematic approach with clinical correlations. Placenta..

[11] Hytten F (1985). Blood volume changes in normal pregnancy. Clin Haematol.

[12] Clark P, Brennand J, Conkie JA, McCall F, Greer IA, Walker ID (1998). Activated protein C sensitivity, protein C, protein S and coagulation in normal pregnancy. Thromb Haemost.

[13] Mosesson MW (2005). Fibrinogen and fibrin structure and functions. J Thromb Haemost.

[14] Mu J, Kanzaki T, Tomimatsu T, Fukuda H, Fujii E, Takeuchi H, et al (2002). Investigation of intraplacental villous arteries by Doppler flow imaging in growth restricted fetuses. Am J Obstet Gynecol.

[15] Chaddha V, Viero S, Huppertz B, Kingdom J (2004). Developmental biology of the placenta and the origins of placental insufficiency. Semin Fetal Neonatal Med.

[16] Greer IA (1998). The special case of venous thromboembolism in pregnancy. Haemostasis.

[17] Hansen AT, Andreasen BH, Salvig JD, Hvas AM (2011). Changes in fibrin D-dimer, fibrinogen, and protein S during pregnancy. Scand J Clin Lab Invest.

[18] Liu J, Yuan E, Lee L (2012). Gestational age-specific reference intervals for routine haemostatic assays during normal pregnancy. Clin Chim Acta.

[19] Francalanci I, Comeglio P, Liotta AA, Cellai AP, Fedi S, Parretti E, et al (1995). D-Dimer in intra-uterine growth retardation and gestational hypertension. Thromb Res.

